# Screening seven hub genes associated with prognosis and immune infiltration in glioblastoma

**DOI:** 10.3389/fgene.2022.924802

**Published:** 2022-08-12

**Authors:** Yesen Zhang, Huasheng Fan, Chun Zou, Feng Wei, Jiwei Sun, Yuchun Shang, Liechun Chen, Xiangyu Wang, Beiquan Hu

**Affiliations:** ^1^ Department of Neurosurgery, The First Affiliated Hospital of Jinan University, Guangzhou, GD, China; ^2^ Department of Neurosurgery, The First Affiliated Hospital of Bengbu Medical College, Bengbu, China; ^3^ Department of Neurosurgery, The Fifth Affiliated Hospital of Guangxi Medical University, Nanning, GX, China; ^4^ Department of Neurology, The Second Affiliated Hospital of Guangxi Medical University, Nanning, GX, China

**Keywords:** glioblastoma, risk score, prognostic biomarkers, microRNAs, immune cell infiltration

## Abstract

Glioblastoma (GBM) is the most common and deadly primary brain tumor in adults. Diagnostic and therapeutic challenges have been raised because of poor prognosis. Gene expression profiles of GBM and normal brain tissue samples from GSE68848, GSE16011, GSE7696, and The Cancer Genome Atlas (TCGA) were downloaded. We identified differentially expressed genes (DEGs) by differential expression analysis and obtained 3,800 intersected DEGs from all datasets. Enrichment analysis revealed that the intersected DEGs were involved in the MAPK and cAMP signaling pathways. We identified seven different modules and 2,856 module genes based on the co-expression analysis. Module genes were used to perform Cox and Kaplan-Meier analysis in TCGA to obtain 91 prognosis-related genes. Subsequently, we constructed a random survival forest model and a multivariate Cox model to identify seven hub genes (KDELR2, DLEU1, PTPRN, SRBD1, CRNDE, HPCAL1, and POLR1E). The seven hub genes were subjected to the risk score and survival analyses. Among these, CRNDE may be a key gene in GBM. A network of prognosis-related genes and the top three differentially expressed microRNAs with the largest fold-change was constructed. Moreover, we found a high infiltration of plasmacytoid dendritic cells and T helper 17 cells in GBM. In conclusion, the seven hub genes were speculated to be potential prognostic biomarkers for guiding immunotherapy and may have significant implications for the diagnosis and treatment of GBM.

## Introduction

Glioblastoma (GBM), a World Health Organization Grade IV glioma (Thakkar et al., 2014), is the most malignant and invasive primary brain tumor in adults (Hoshide and Jandial, 2016). The National Cancer Institute reported that GBM accounts for 52% of all primary tumors of brain in the United States (de Robles et al., 2015). The overall survival time is less than a year from the diagnosed date in most GBM patients if left untreated (Laws et al., 2003; Mirimanoff et al., 2006). Advanced age is the most common risk factor and prognostic factor associated with a cancer diagnosis (Yancik et al., 1998; Yancik et al., 2001). However, the basis for the increased incidence of GBM among elderly individuals is poorly understood and underexplored (Kim et al., 2021). Moreover, leptomeningeal spread is one of the most severe complications of GBM, along with other complications, such as intratumor hemorrhage, status epilepticus, and hydrocephalus (Mandel et al., 2014; Noh et al., 2015; Autran et al., 2019). At present, the standard diagnostic method of standard therapy consists of surgery along with chemotherapy and radiation, which insignificantly improved GBM patient’s survival (Stupp et al., 2005). Furthermore, due to the risk of invasion and high cost, a better diagnosis method is needed.

MicroRNAs (miRNAs) are small non-coding RNAs with a total length of 18–25 nucleotides (Chen and Rajewsky, 2007) that control diverse cellular and developmental events by repressing large sets of target mRNAs (Ding et al., 2009). MiRNAs can inhibit the translation of downstream target mRNA to regulate both physiological and pathological processes (Inui et al., 2010). The dysregulation of miRNAs contributes to cell growth, migration, proliferation, invasion, and metastasis in glioblastoma (Que et al., 2015). Studies have demonstrated an association between glioblastomas and miRNAs. Some miRNAs are gene silencers of anti-apoptotic genes that inhibit the growth and survival of glioblastoma. The miRNA signature can be used for the diagnosis of brain tumors (Ahmed et al., 2021). Having some studied showed that high expression of RBM8A (Lin et al., 2021; Wei et al., 2022) and MDK (Hu et al., 2020; Hu et al., 2021) promotes growth and migration and showed a good diagnostic and prognostic value in GBM. Moreover, EN1 and EGR3 may have predictive prognostic value for GBM (Qin et al., 2020), however, the biomarkers of GBM still need to be further explored.

Furthermore, studies have shown that the use of molecular diagnostics and gene analysis in GBM has increased (Louis et al., 2021). In the present study, we used GBM and control brain tissues from the Cancer Genome Atlas (TCGA) and the Gene Expression Omnibus (GEO) databases to perform bioinformatics analysis. The purpose of this study was to identify relevant potential markers using a diagnostic classifier to treat patients with GBM and to construct a risk score model to improve accuracy.

## Materials and methods

### Data preprocessing

Four datasets, TCGA, GSE68848, GSE16011, and GSE7696, included 729 GBM and 45 normal brain tissue samples. RNA sequencing and the corresponding clinical information for 145 GBM patients and five healthy (control) individuals were downloaded from TCGA (https://portal.gdc.cancer.gov/). Expression profiles of the GSE68848, GSE16011, GSE7696, and GSE25631 datasets were downloaded from the GEO (https://www.ncbi.nlm.nih.gov/geo/). The GSE68848 expression profile contained 580 samples, including 228 GBM tumors and 28 control tissues, detected by the Affymetrix Human Genome U133 Plus 2.0 Array based on the GPL570 platform (Madhavan et al., 2009). Meanwhile, astrocytoma samples were removed. GSE16011 contained 284 clinical samples, including 276 glioma samples of all histology and eight control tissue samples, detected by the Affymetrix GeneChip Human Genome U133 Plus 2.0 Array based on the GPL8542 platform (Gravendeel et al., 2009). A total of 84 samples of GSE7696 contained 80 GBM specimens from patients treated in clinical trials and four samples of normal brain tissue (non-tumoral), detected using the Affymetrix Human Genome U133 Plus 2.0 Array based on the GPL570 platform (Murat et al., 2008; Lambiv et al., 2011). Gene expression profiles of GSE68848, GSE16011, and GSE7696 were normalized using the “RMA” function in the Affy package. The “varianceStabilizingTransformation” function of the DESeq2 package (Love et al., 2014) was used to normalize the TCGA expression profile.

GSE25631 contains 82 surgical specimens of primary glioblastoma multiform and five normal brain tissues from areas surrounding arteriovenous malformations (AVM) as a control, detected by Illumina Human v2 miRNA expression beadchip based on GPL8179 (Chen et al., 2012). Expression profile of GSE25631 was used to normalize by the “lumiExpresso” function in the lumi R package.

### Differential expression and enrichment analyses

Differential expression analysis was performed for expression profiles in three datasets (GSE68848, GSE16011, and GSE7696) between GBM patients and controls using the limma R package (Ritchie et al., 2015). We obtained differentially expressed genes (DEGs) from TCGA dataset using the DESeq2 R package (Love et al., 2014). A adjust *p*-value of <0.05 was set as the screening criterion to obtain DEGs. Intersections of DEGs were obtained from four datasets (TCGA, GSE68848, GSE16011, and GSE7696), and the upregulated or downregulated DEGs were identified using the ggVennDiagram package (Gao et al., 2021). Furthermore, Gene Ontology (GO) analysis can be classified into different gene functions, including biological process (BP), molecular function (MF), and cellular component (CC). The Kyoto Encyclopedia of Genes and Genomes (KEGG) stores many pathways and is widely used. Intersected DEGs were used to construct GO and KEGG analyses using the clusterProfile R package (Yu et al., 2012). Biological functions and pathways of intersected DEGs with a *p* value of <0.05 were considered statistically significant.

### Gene set enrichment analysis

Gene set enrichment analysis (GSEA) revealed underlying pathways and evaluated microarray data at the gene set level (Subramanian et al., 2005). The expression profile of TCGA was used to perform GSEA using the clusterProfiler R package.

### Co-expression analysis of intersected DEGs

Weighted gene co-expression network analysis (WGCNA) is a systems biology method for clustering genes identified by similar expression patterns and transforming the expression of genes into modules (Langfelder and Horvath, 2008). The WGCNA R software package (Langfelder and Horvath, 2008) was used to construct a co-expression network to obtain module genes using the intersected DEGs. To ensure a scale-free topology, a soft threshold function was applied to calculate the power parameters. Dynamic tree cutting was used to identify co-expressed gene modules and establish a hierarchical clustering tree based on the unsigned topological overlap matrix (TOM)-based on dissimilarity. Genes with similar expression profiles were grouped into the network modules. Pearson’s correlation analysis was performed to calculate the module-trait correlation.

### Construction of random survival forest model

Module genes were used to perform Cox and Kaplan-Meier analyses in TCGA to obtain prognosis-related genes. Additionally, the Metascape network tool was used to perform functional enrichment analysis of prognosis-related genes. The random forest survival model was performed with prognosis-related genes using the “coxph” function of the survival package. We used the *randomForestSRC* package in R to rank the prognosis-related genes, and those with a relative importance of >0.4 were considered as final hub genes. Furthermore, the expression levels between the GBM and normal samples of hub genes were shown using a violin plot and heat map in TCGA.

### Risk score prediction based on Cox regression mode

A risk score model was established by multivariate Cox regression analysis using hub genes to test independent prognostic value. GBM patients were divided into groups between high-risk and low-risk based on the median of the risk score to obtain the survival time of patients by “predict” function in the survival R package (Barakat et al., 2019). The forestplot R package determined the hazard ratio (HR) and 95% confidence interval (CI) of each hub gene variable. The median risk score was used to predict 1- and 2-year survival time by time-dependent receiver operating characteristic (ROC) curves in patients with GBM. The nomogram was used for diagnosis and prognosis (Iasonos et al., 2008; Fu et al., 2017), consisting of hub genes to estimate the prognosis probability at 1 and 2 years using the rms package. Subsequently, calibration plots were used to evaluate the calibration of the nomograms. The area under the curve (AUC) values of these hub genes were calculated separately in the four datasets using the pROC R package.

### Regulation of miRNA-mRNA

Differential expression analysis was used to identify the upregulated and downregulated differentially expressed miRNAs (DEmiRs) in GSE25631 using the limma package. Target genes of the top three DEmiRs with the largest |log(fold change)| and the binding sites of the top three DEmiRs and hub genes were predicted using the TargetScan (http://www.targetscan.org/vert_72) database.

### Landscape of immune cell infiltration

The single-sample gene set enrichment analysis (ssGSEA) algorithm was performed based on 24 immune cell types to comprehensively assess the immunologic characteristics of every sample using the GSVA R packages. The degree of immune cell infiltration between GBM and control samples was calculated using the limma R package. Pearson’s correlation was used to calculate the correlation between hub genes and immune cells. Moreover, we used the CIBERSORT algorithm to assess the distribution of 22 immune cell types in each TCGA sample.

## Results

### Biological function of DEGs

**FIGURE 1:**
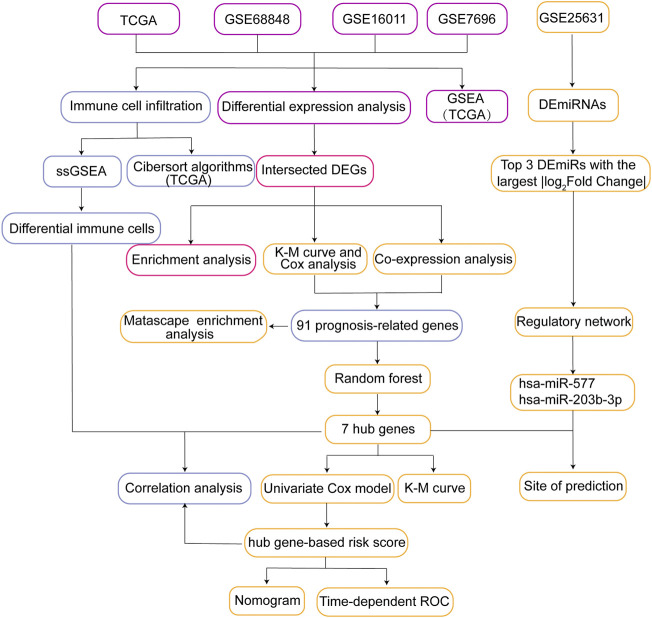
Flow chart of the present study. DEGs, differentially expressed genes; ssGSEA, single sample Gene Set Enrichment Analysis; GSEA, Gene Set Enrichment Analysis; K-M curve, Kaplan-Meier curve; Time-dependent ROC, Time-dependent receiver operating characteristic curve; TCGA, The Cancer Genome Atlas.

**FIGURE 2:**
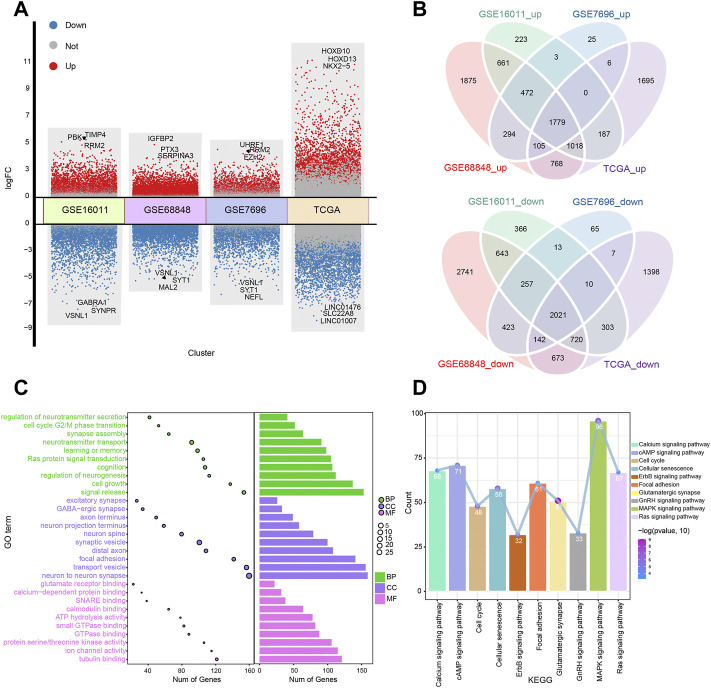
Differential expression analysis and enrichment analysis in glioblastoma (GBM). **(A)** Volcano plots of differentially expressed genes (DEGs) in four datasets (GSE16011, GSE68848, GSE7696, and TCGA). Red dots indicate upregulated DEGs, grey dots indicate not-regulated DEGs, and blue dots indicate downregulated DEGs. **(B)** Venn diagram of the intersected DEGs of four datasets, including commonly up/downregulated DEGs. **(C)** BP, MF, and CC were obtained by Gene Ontology enrichment analysis. BP, biological process; MF, molecular function; CC, cellular component. **(D)** Functional pathways of intersected DEGs.

A flowchart of the study is presented in Figure 1. We identified DEGs between GBM patients and controls to identify dysfunctional genes associated with GBM (Figure 2A). A total of 8,676 DEGs were identified in GSE16011, 14,592 DEGs in GSE68848, 5,622 DEGs in GSE7696, and 14,003 DEGs in TCGA. Among these DEGs, 3,800 intersected DEGs had the same direction of differential expression, including 1779 upregulated and 2021 downregulated DEGs in all four datasets (Figure 2B). We analyzed intersected DEGs for GO enrichment analysis and the results showed the following: 1) in BP, intersected DEGs were mainly involved in neurotransmitter transport and cognition; 2) in CC, intersected DEGs were mainly involved in synaptic vesicles and neuron-to-neuron synapses; and 3) in MF, intersected DEGs were mainly enriched in tubulin binding and GTPase binding (Figure 2C). Furthermore, we analyzed these intersected DEGs in the KEGG pathway. The results showed that intersected DEGs were mainly involved in the MAPK, cAMP, and Ras signaling pathways (Figure 2D). Results remind that intersected DEGs were involved in biological functions and may be promote the development of GBM.

### Identification of module genes in GBM

**FIGURE 3:**
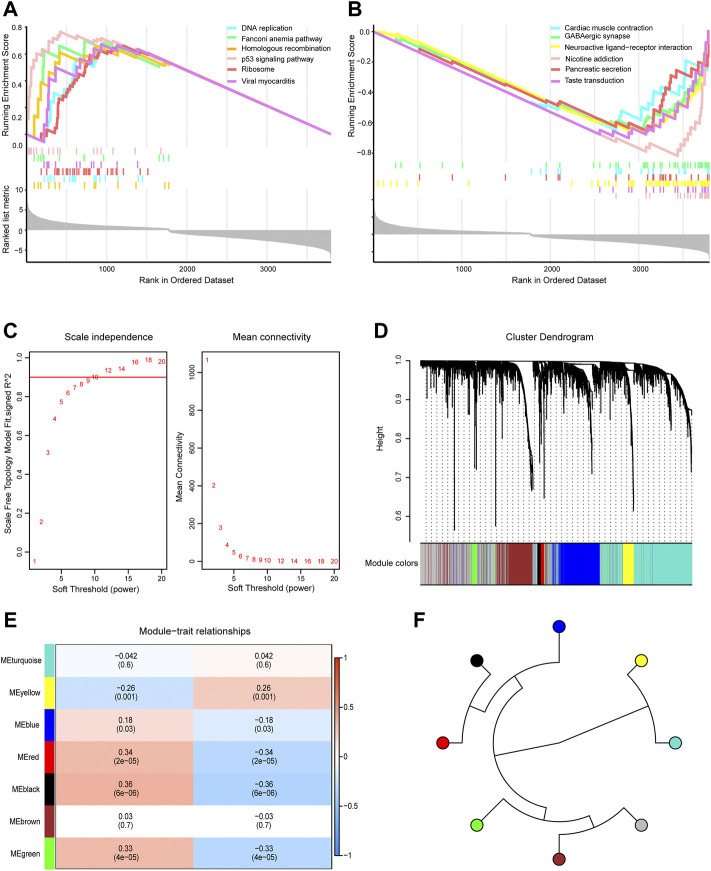
Gene Set Enrichment Analysis (GSEA) and Weighted Gene Co-expression Network Analysis. **(A)** GSEA showed head of six pathways enriched in glioblastoma (GBM) patients. **(B)** GSEA showed tail of six pathways enriched in GBM patients. **(C)** Network topology analysis under different soft threshold power. **(D)** Cluster tree showed co-expression modules based on WGCNA. **(E)** Correlation between seven key modules and clinical traits. **(F)** Correlation between the modules in GBM.

GSEA showed that genes in the TCGA dataset were positively correlated with the P53 signaling pathway and DNA replication (Figure 3A) which may be promote the development of GBM and negatively correlated with GABAergic synapses and nicotine addiction (Figure 3B) that may be inhibit the development of GBM. To screen the key module most associated with GBM, WGCNA was performed using intersected DEGs. The lowest power that gave an independence larger than 0.90 was 10 (Figure 3C). A total of 3,800 intersected DEGs with similar expression patterns were placed into seven different modules to obtained 2,856 module genes (Figures 3D,E). As shown in Figure 3F, we obtained the correlation between seven different modules in GBM.

### Identification of optimal diagnostic gene biomarkers

**FIGURE 4:**
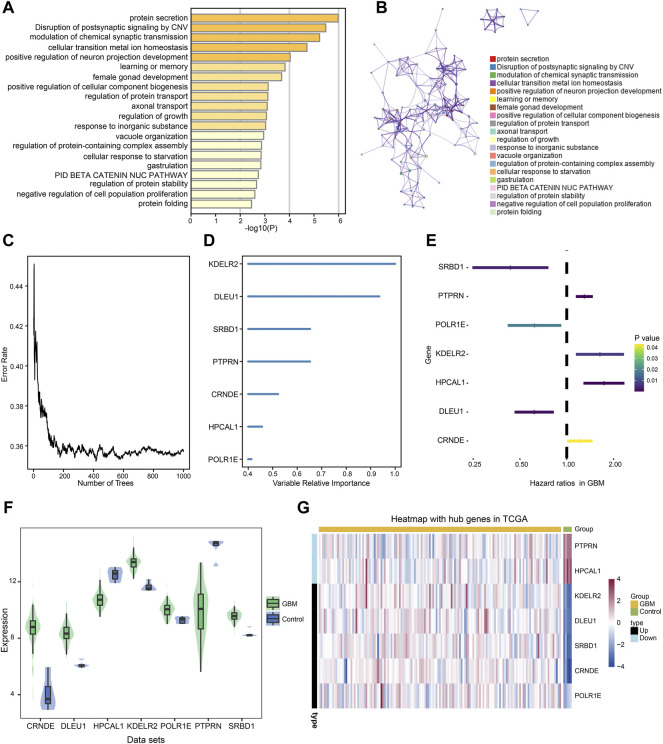
Metascape enrichment analysis of 91 prognosis-related genes and identified the hub genes in glioblastoma (GBM). **(A)** Gene Ontology (GO) terms and Kyoto Encyclopedia of Genes and Genomes (KEGG) pathways involved in 91 prognosis-related genes. **(B)** The interactions of these GO terms and KEGG pathways. **(C)** Relationship between error rate and number of trees. **(D)** Random forest model was performed to determine the importance of the seven genes. **(E)** Forest plot of the multivariate Cox regression analysis in prognosis-related genes. The genes with a hazard ratio (HR) >1 were identified as associated with prognostic risk factors, an HR <1 was considered a protective factor. **(F)** Violin plot showed the expression of seven hub genes in control and GBM samples. The thick black bar in the middle indicates the interquartile range, and the black line extending from it represents the 95% confidence interval. **(G)** Heat map showed the expression of seven hub genes in GBM and control samples in TCGA dataset. GBM, glioblastoma; TCGA, The Cancer Genome Atlas.

Intersected DEGs were used to identify prognosis-related genes in the four datasets using Cox regression and Kaplan-Meier curve analyses in TCGA. Subsequently, 91 prognosis-related genes were identified. Metascape enrichment analysis of the 91 prognosis-related genes indicated protein secretion and disruption of postsynaptic signaling by CNV (Figure 4A). Figure 4B shows the interactions between these GO terms and the KEGG pathways. The relationship between the error rate for the data and the number of classification trees (Figure 4C), and the importance of the seven genes (DLEU1 and POLR1E, SRBD1, PTPRN, KDELR2, HPCAL1, and CRNDE) by Random forest model are shown in Figure 4D. In the multivariate Cox regression analysis, PTPRN, KDELR2, HPCAL1, and CRNDE were high-risk factors (Figure 4E), in which KDELR2 and CRNDE were overexpressed in GBM (Figure 4F). Heat maps showed the expression of seven hub genes, including five upregulated and two downregulated genes, in TCGA (Figure 4G). In addition, the 1-year survival time of patients showed the DLEU1 and POLR1E groups had a significantly better overall survival (Supplementary Figure S1).

### Prognostic risk score as a prognostic tool in GBM

**FIGURE 5:**
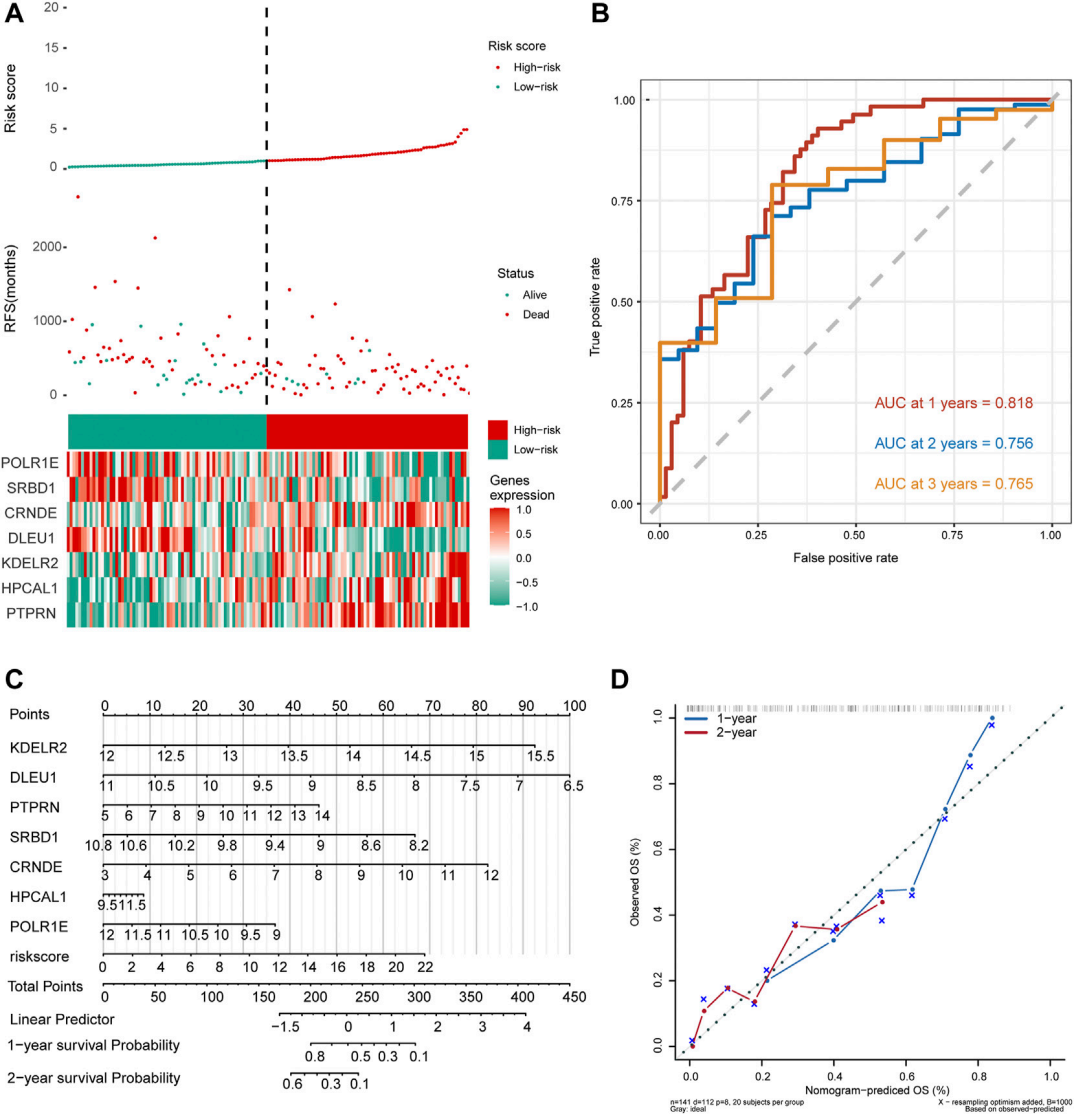
Seven hub gene-risk scores were constructed based on the median risk score. **(A)** Distribution of glioblastoma (GBM) patients and survival states of GBM patients with the risk scores, heat-map of seven hub genes. **(B)** Time-dependent receiver operating characteristic (ROC) analysis in The Cancer Genome Atlas for median of risk score in GBM. AUC, area under the curve. **(C)** The nomogram was built by seven hub genes. **(D)** The calibration plots for internal validation of the nomogram for 1- and 2-year survival. OS, overall survival.

As shown in Figure 5A, the number of deaths in the high-risk group was significantly higher than that in the low-risk group in the risk-score model. Time-dependent ROC curve analysis for the median risk score was used to show that the AUC values for 1-, 2-, and 3-year survival were 0.818, 0.756, and 0.765, respectively (Figure 5B). We then constructed a nomogram model for hub genes and risk scores to predict the 1- and 2-year overall survival probability of GBM patients (Figure 5C). Subsequently, we used calibration plots of the nomogram to estimate the 1- and 2-year overall survival probabilities (Figure 5D). In conclusion, seven hub genes were predicted significantly associated with overall survival probability of GBM patients.

### Regulation between DEmiRs and DEGs

**FIGURE 6:**
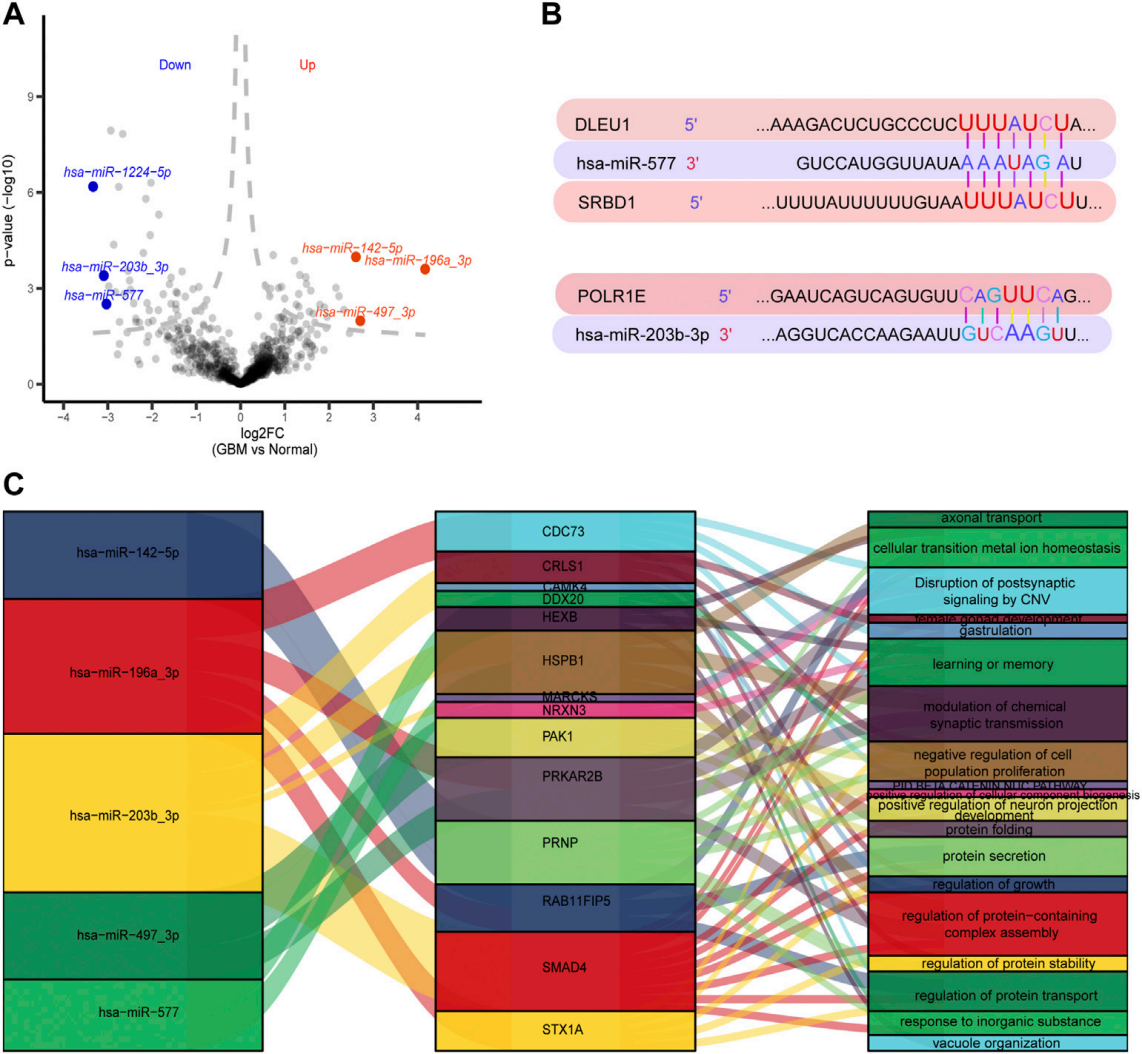
Regulation between 91 prognosis-related genes and the top three differentially expressed miRNAs (DEmiRs) with the largest |log_2_ (fold change)|. **(A)** Volcano plots of DEmiRs in GSE25631. Red dots indicate the top three up-regulated DEmiRs and blue dots indicate top three down-regulated DEmiRs. **(B)** Binding sites of top three DEmiRs with the largest |log_2_ (fold change)| and prognosis-related genes. **(C)** A Sankey diagram was generated using three DEmiRs with the largest |log_2_ (fold change)|, prognosis-related genes and pathways.

A total of 147 DEmiRs were identified in GSE25631, containing 65 upregulated and 82 downregulated DEGs between GBM and control samples, by differential expression analysis (Figure 6A). We further explored the regulation between the top three DEmiRs with the largest |log_2_ (fold change)| with 91 prognosis-related genes in GBM patients. The TargetScanHuman database showed the binding sites of two miRNAs (miR-577 and miR-203b-3p) on long noncoding RNA (lncRNA) (DLEU1) and two mRNAs (POLR1E and SRBD1). The results showed that position 1,622–1,629 of the DLEU1 3′ UTR was bound to miR-577, position 278–284 of the SRBD1 3′ UTR was bound to miR-577, and position 934–940 of the POLR1E 3′ UTR was bound to hsa-miR-203b-3p (Figure 6B). We established a regulatory network involving five DEmiRs that targeted prognosis-related genes (Figure 6C).

### Exploring the infiltration of immune cell types in GBM and control tissues

**FIGURE 7:**
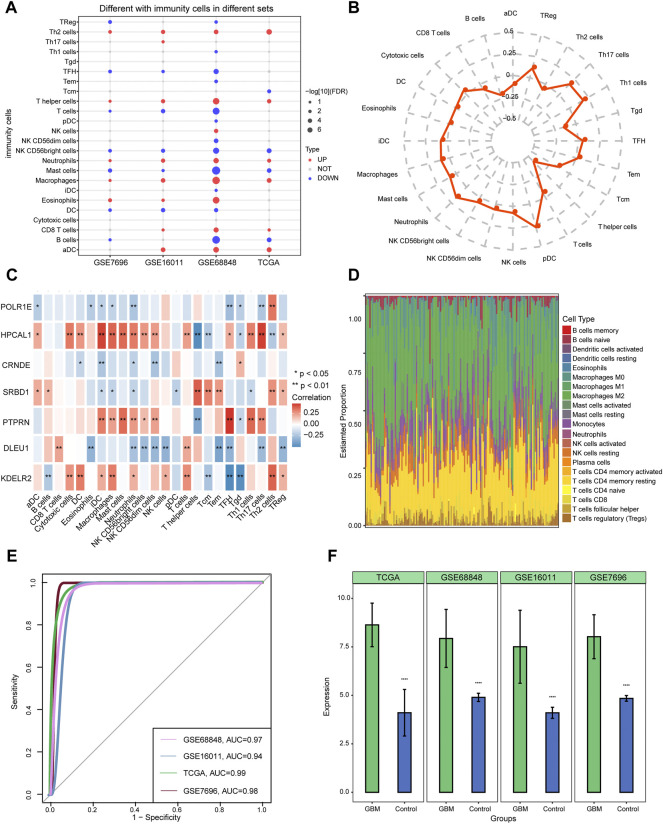
Identifying immune cell infiltration in glioblastoma (GBM) and expression of CRNDE in four datasets. **(A)** Twenty-four immune cell types were used to constructed ssGSEA in four datasets. Red dots indicate the high-infiltrated immune cell types and blue dots indicate low-infiltrated immune cell types. **(B)** Radar chart shows the correlation between immune cells and seven hub gene-risk score. **(C)** Pearson’s correlation shows the correlation between immune cells and seven hub genes. **(D)** Distribution of 22 immune cell types in each sample from TCGA using the CIBERSORT tool. **(E)** The area under the curves (AUCs) of CRNDE in four datasets. **(F)** The expression of CRNDE between GBM and controls. ****p < 0.001. GBM, glioblastoma.

Four datasets (GSE68848, GSE16011, GSE7696, and TCGA) were selected to study immune cell infiltration. Macrophages, neutrophils, and T helper cells showed a higher degree of infiltration in GBM than in controls (Figure 7A). Plasmacytoid dendritic cells (pDCs) and T helper (Th) 17 cells were found to have a significantly high correlation, while T helper cells showed a low correlation between 24 immune cell types and the median risk score (Figure 7B). The results of correlation with immune cell types showed that POLR1E, DLEU1, and CRNDE were negatively correlated with most immune cell types, whereas HPCAL1, PTPRN, SRBD1, and KDELR2 were positively correlated with most immune cells (Figure 7C). Furthermore, among the 22 immune cell types, macrophages M2 showed high infiltration between GBM and control samples from TCGA (Figure 7D). Evaluation of the involvement of CRNDE in GBM indicated an AUC of 0.94 in GSE16011, and the AUCs were more than 0.97 in the GSE68848, GSE7696, and TCGA datasets (Figure 7E). CRNDE was highly expressed in GBM compared with the control samples in the four datasets and is shown in the bar diagram (Figure 7F).

## Discussion

Patients with GBM have poor prognosis (Gregucci et al., 2021) and novel diagnosis and treatment approaches against GBM are urgently needed. In this study, co-expression network and univariate Cox regression analyses were constructed to identify seven hub genes related to prognosis, including KDELR2, DLEU1, PTPRN, SRBD1, CRNDE, HPCAL1, and POLR1E. Among which, we found that PTPRN, KDELR2, HPCAL1, and CRNDE were high-risk factors by the nomogram model and survival prediction. Furthermore, the seven hub genes were associated with immune cell infiltration. In short, immune-related genes may subsequently affect prognosis of GBM patients by affecting the abundance of infiltrating cells in biological processes. Moreover, we identified gene-related prognosis in the regulation of miRNAs and that involved in biological functions to promote the occurrence and development in patients with GBM.

By comparing GBM tissues with normal tissues in the four datasets, we identified the intersected DEGs to explore new therapeutic targets and prognostic markers of GBM. Enrichment analysis revealed that the intersected DEGs of the four datasets were involved in the Ras (Sun et al., 2021), MAPK, and cAMP signaling pathways (Li et al., 2021). The MAPK signaling pathway is a major RAS effector pathway that plays an important role in the survival, drug resistance, and metastasis of cancer (Reardon et al., 1999; Roberts and Der, 2007; Santarpia et al., 2012). We explored the potential of 91 prognosis-related genes as prognostic markers for GBM patients. The ribosome and calcium (Ca^2+^) signaling pathway is activated in GBM, with inhibition of GABAergic synapses and nicotine addiction. GBM cells induce and acquire stem cell-like cell (GSCs) properties *via* exogenous ribosomes, which may promote the development of GSCs in GBM tissues (Shirakawa et al., 2021). Recent studies have indicated that the Ca^2+^ signaling pathway mainly affects the gene expression and invasion of GBM cells (So et al., 2021). Additionally, dysfunction of GABAergic damage plays an important role in neurodevelopmental disorders (Tang et al., 2021). Nicotine protects against psychiatric symptoms in schizophrenia (Lucatch et al., 2018), suggesting that GABAergic synapses and nicotine addiction may play protective roles. Furthermore, intersected DEGs for GO enrichment analysis, which involved in cognition and GTPase binding. The Rho family of small GTPases, which could regulate the invasion and migration of GBM cells (Al-Koussa et al., 2020). There are also studies revealing that cognition is significantly associated with survival of diagnosis and relapse in GBM patients (Johnson and Wefel, 2013).

WGCNA, used for finding modules of highly correlated genes which enhanced the functions for co-expression network analysis (Langfelder and Horvath, 2008). Moreover, WGCNA has been used to analyze gene expression data from lung cancer (Ding et al., 2019), medulloblastoma (Du et al., 2020), colon adenocarcinoma (Wang et al., 2020). In this study, we distinguished seven modules and 2,856 module genes in GBM using WGCNA. We identified seven modules, yellow, blue, turquoise, black, red, green, and brown, whose genes are strongly related to GBM. Among these, module genes play a positive role in GBM. Therefore, our results indicate that complex gene networks regulate GBM occurrence and development.

According to our findings in the current study, the results of the random forest survival model showed that KDELR2, DLEU1, PTPRN, SRBD1, CRNDE, HPCAL1, and POLR1E may be hub genes significantly associated with survival in GBM. KDELR2 is a novel biomarker that is highly expressed in high-grade gliomas (Mao et al., 2020). Upregulated DLEU1 was discovered in GBM tissues and might play an essential role in accelerating GBM development by modulating cell proliferation and apoptosis (Liu et al., 2019). Furthermore, PTPRN has been used for prognosis and plays a role in treating patients with radiotherapy and chemotherapy (Zhu et al., 2021). A previous study showed that the expression of lncRNAs CRNDE was high in patient tissues and was associated with poor prognosis in GBM (Zhao et al., 2021). miR-577 was found to have binding sites for DLEU1 and SRBD1 by regulated network analysis. DLEU1 and SRBD1 may be the target genes of miR-577. Furthermore, POLR1E expression was negatively modulated by miR-203b-3p expression. It is notable that few studies on SRBD1, HPCAL1, and POLR1E have been reported in GBM, while SRBD1 and POLR1E hardly appear in the literature.

The results showed that the low/high-risk score was related to OS by a risk score system, suggesting that the risk factors were correlated with the prognosis of GBM patients. Compared to the high-risk group, the survival time of GBM patients in the low-risk group was longer. Time-dependent ROC analysis showed that the hub genes (KDELR2, DLEU1, PTPRN, SRBD1, CRNDE, HPCAL1, and POLR1E) had good performance in survival prediction. Furthermore, the nomogram model visualizes the influence of risk factors and survival prediction to indicate CRNDE as a poor gene of prognosis. AUC analysis showed that AUCs of CRNDE were all greater than 0.94 in four datasets, the results certified the gene had a high accuracy of the diagnostic models. Above all, overexpression of CRNDE may be a key gene involved in the prognosis of patients with GBM.

Using ssGSEA, pDCs, and Th17 cells were found to have a significantly high degree of infiltration between hub genes and immune cells. Recent studies have indicated that pDCs lead to immunosuppression and promote tumor growth (Zhou et al., 2021). Moreover, the results of Pearson analysis showed that HPCAL1 and PTPRN had high infiltration in Th17 cells, POLR1E, SRBD1, DLEU1, KDELR2, and CRNDE had high infiltration in Th2 cells. Th17 cell populations have been implicated in the liver, ovaries, breast, melanoma, and colon (Zou and Restifo, 2010). Removal of the tumour from cancer patients that becoming less polarised towards Th2 cell (Tatsumi et al., 2002), reminding us that Th2 cell may be consist in cancers. pDCs and Th17 cells are promising targets for GBM immunotherapy. However, the Th2 and Th17 cell subsets may be weakly correlated in GBM (Miller and Weinmann, 2009) that we need to further study. In addition, in the proportion of 22 immune cell types, macrophages M2 had high infiltration between GBM and control samples of TCGA. Previous studies have found that M2 macrophages participate in glioma progression and their prognostic value in gliomas has been affirmed (Zhang et al., 2021).

However, this study had some limitations. First, all the data analyzed were from the GEO and TCGA cohorts, and the experiments were not validated. Second, studies with large sample sizes are warranted to validate our findings. Functional analysis of hub genes involved in immunoregulation requires further research.

## Conclusion

We constructed diagnostic models to identify seven hub genes related to prognosis in GBM, including KDELR2, DLEU1, PTPRN, SRBD1, CRNDE, HPCAL1, and POLR1E. Subsequently, pDC and Th17 cells were found to have a significantly high degree of infiltration, with a risk score of seven hub genes. In conclusion, our study provides novel targets to improve the treatment efficacy and prognostic accuracy of GBM.

## Data Availability

The original contribution presented in the study are included in the article/Supplementary Material, further inquiries can be directed to the corresponding authors.
