# Supplementary material for: Screening seven hub genes associated with prognosis and immune infiltration in glioblastoma
Source: Front Genet. 2022 Aug 12;13:924802. doi: 10.3389/fgene.2022.924802 (PMC9412194; doi:10.3389/fgene.2022.924802)
Supplement: Supplementary file 1 [file DataSheet1.PDF]

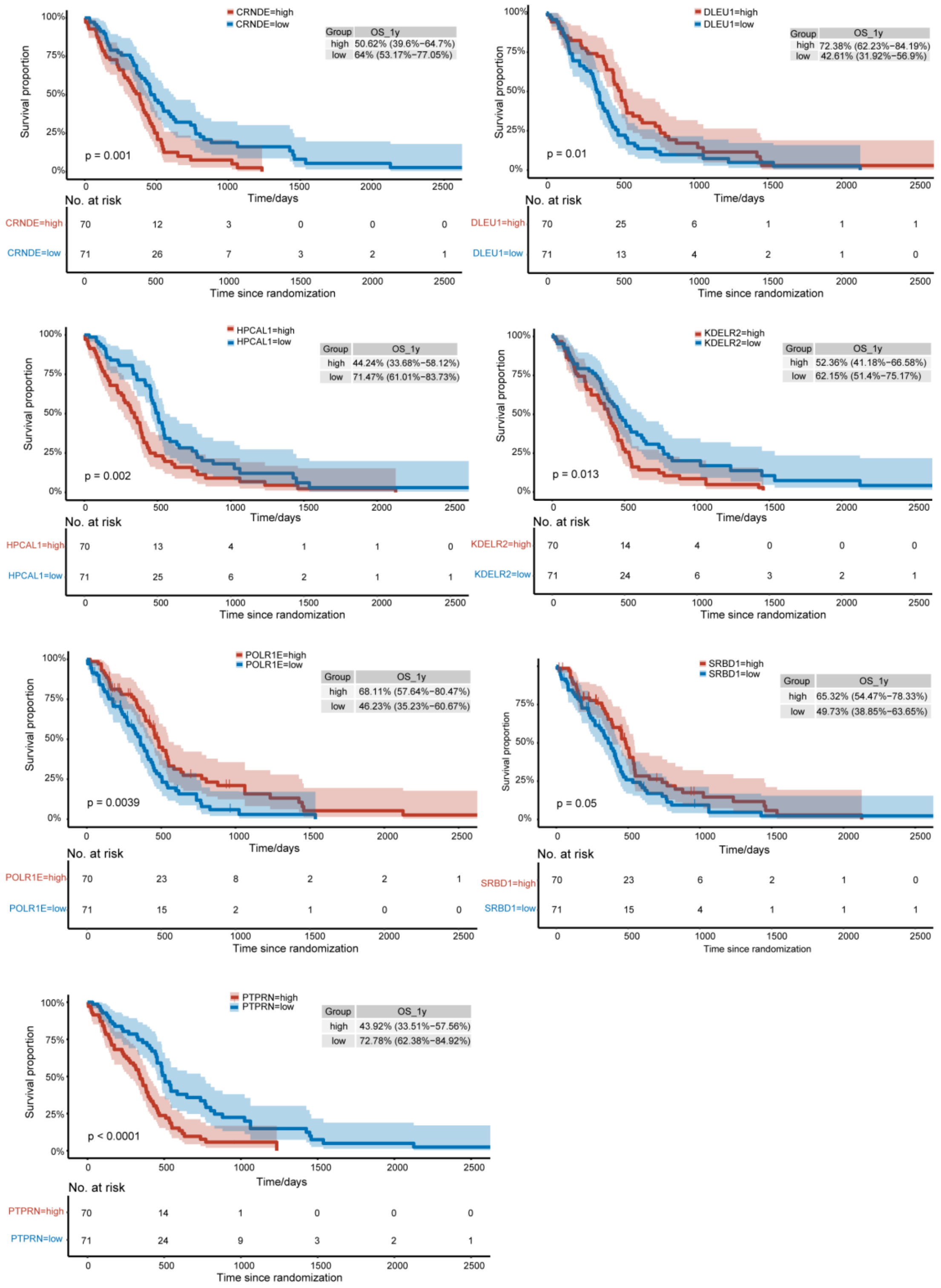

Figure S1 Kaplan-Meier survival curve of glioblastoma patients stratified by low/high expression of the seven hub genes.
